# The impact of microcarrier culture optimization on the glycosylation profile of a monoclonal antibody

**DOI:** 10.1186/2193-1801-2-25

**Published:** 2013-01-28

**Authors:** Ana Rita Costa, Joanne Withers, Maria Elisa Rodrigues, Niaobh McLoughlin, Mariana Henriques, Rosário Oliveira, Pauline M Rudd, Joana Azeredo

**Affiliations:** 1 IBB-Institute for Biotechnology and Bioengineering, Centre of Biological Engineering, University of Minho, Campus de Gualtar, 4710-057 Braga, Portugal; 2 NIBRT Dublin-Oxford Glycobiology Laboratory, National Institute for Bioprocessing Research and Training, Fosters Avenue, Mount Merrion, Blackrock, Co, Dublin, Ireland

**Keywords:** Microcarrier, Cytodex 3, Glycosylation, Monoclonal antibody, Chinese hamster ovary

## Abstract

Microcarriers are widely used for the large-scale culture of attachment-dependent cells with increased cell densities and, ultimately, higher product yield. In these processes, the specific culture conditions can affect the quality of the product, which is closely related to its glycosylation pattern. Furthermore, the lack of studies in the area reinforces the need to better understand the effects of microcarrier culture in product glycosylation. Consequently, in this work, the glycosylation profile of a monoclonal antibody (mAb) produced by adherent CHO-K1 cells grown in Cytodex 3 was evaluated under different conditions, and compared to that obtained of typical adherent cultures. It was found that microcarrier cultures result in a glycosylation profile with different characteristics from T-flask cultures, with a general increase in galactosylation and decrease in fucosylation levels, both with a potentially positive impact on mAb activity. Sialylation also varied but without a general tendency. This study then showed that the specific culture conditions used in microcarrier culture influence the mAb glycan profile, and each functional element (galactose, core fucose, sialic acid) is independently affected by these conditions. In particular, great reductions of fucosylation (from 79 to 55%) were obtained when using half volume at inoculation, and notable decreases in sialylation (from 23 to 2%) and glycoform heterogeneity (from 20 to 11 glycoforms) were observed for shake flask culture, potentially associated with the improved cell densities achieved in these culture vessels.

## Background

Microcarrier systems are the most well-known technology for the large-scale culture of adherent cells in biotechnology (Chu and Robinson 2001; Kong et al. 1999; Ziao et al. 2002). Indeed, several types of microcarriers have been successfully used for the production of viral vaccines (Berry et al. 1999; Butler et al. 2000; Mendonça et al. 1993; Wu and Huang 2002; Wu et al. 2002) and recombinant proteins (Blüml 2007; Cosgrove et al. 1995; Hu et al. 2000; Wang et al. 2002; Wang and Ouyang 1999), in consequence of an improved surface area available for cell adhesion that results in higher cell density and product yield (Blüml 2007; Hirtenstein et al. 1980; Rudolph et al. 2008; Schürch et al. 1992; Van Wezel 1967). Generally, microcarriers can be divided into microporous (e.g. Cytodex) and macroporous (e.g. CultiSpher, Cytoline, Cytopore), according to the size of the porous, which allow cell growth on the surface or on both surface and inner spaces, respectively (Almgren et al. 1991; Butler 1996; Nilsson et al. 1986; Ozturk and Hu 2006; Spearman et al. 2005; Yokomizo et al. 2004).

The characteristics of the microcarriers are important for the success of the culture (Butler 1996; Ozturk and Hu 2006), but other factors must be considered, such as the size and type of culture vessel (Wang et al. 2002; Wu et al. 1998), and the culture environment (Nam et al. 2008; Ng et al. 1996). Furthermore, it is also important to recognize that the specific conditions of the culture can influence product quality, through effects on protein glycosylation.

Indeed, it has become evident that glycosylation plays critical roles in protein effectiveness *in vivo* (Jenkins and Curling 1994), and the ability to perform this post-translational modification is a major reason for the current choice of mammalian cells as hosts for recombinant therapeutic protein production (Nam et al. 2008).

It is therefore essential to ensure that glycosylation of recombinant proteins in microcarrier culture is consistent with the desired product (Spearman et al. 2005). However, to date, there are limited studies examining the effects of changing between adherent, suspension, and microcarrier cultures on glycosylation of the recombinant product (Nam et al. 2008). Furthermore, the few studies performed typically compare microcarrier culture to culture in suspension (Hooker et al. 2007; Wang et al. 2002; Watson et al. 1994), and not to the normal adherent cell culture conditions, and have shown results that seem to be specific to the type of cell, product, and microcarrier used. For example, increased sialylation was found in Cytodex culture for recombinant human tissue kallikrein production (Watson et al. 1994) and in Cytoline culture of Chinese hamster ovary (CHO) cells in a fluidized bed bioreactor for interferon-γ production (Hooker et al. 2007), while no differences were found in human recombinant erythropoietin (EPO) produced by CHO cells in fluidized bed bioreactor cultures with Cytoline (Wang et al. 2002).

In an effort to elucidate the effects of microcarrier culture on protein glycosylation, the present work evaluates the glycan profile of a monoclonal antibody (mAb), with application on cancer therapy, produced by CHO-K1 cells grown in microcarriers. Different culture conditions are assessed to determine their influence on mAb glycosylation, particularly on the galactosylation, fucosylation and sialylation levels, with discussion of their potential impact on the biological effectiveness of the mAb. Additionally, the glycosylation profiles obtained in microcarrier cultures are compared to those of adherent culture in common culture vessels (T-flasks).

## Results and discussion

**Figure 1:**
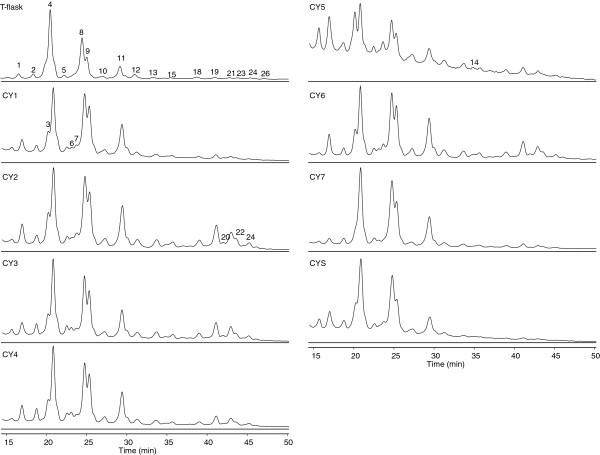
**N-glycosylation profile of the monoclonal antibody produced by CHO-K1 cells cultured under different culture conditions: T-flask; CYi – Cytodex 3 in vented conical tubes; CYS – Cytodex 3 in shake flask.**

An appropriate glycosylation of recombinant proteins, particularly mAbs, is important for their biological activity and clinical efficacy (Spearman et al. 2005). It is therefore essential to ensure that glycosylation in microcarrier cultures is consistent with the desired product. In this study, mAb-producing CHO-K1 cells were cultured in microporous Cytodex 3 carriers, under different culture conditions to evaluate their impact on the glycosylation profile of the mAb. Additionally, the glycosylation obtained in microcarrier cultures was compared to that of normal adherent culture conditions in T-flasks. The profiles obtained by normal phase HPLC are shown in Figure 1, and the glucose unit (GU) values and tentative structure assignments of the peaks identified, as well as the relative (%) peak area obtained in each assay, are shown in Table 1. Glycans found in all conditions are mainly complex biantennary structures with a high degree of heterogeneity, containing different terminal sugars, including sialic acid (S), galactose (G), N-acetylglucosamine (A) and core fucose (F). However, differences can be found between the microcarrier cultures and the typical adherent culture in T-flask in the prevalence of certain glycans, particularly of the most typical IgG1 structures: FA2 (peak 4), FA2G1 (peaks 8 and 9, corresponding to α1-6 and α1-3-linked fucose, respectively), and FA2G2 (peak 11). Indeed, FA2 and FA2G1 (α1-6) show a general tendency to decrease, while FA2G1 (α1-3) and FA2G2 levels increase in microcarrier cultures.

**Table 1:** **Data of mean glucose unit (GU) value, tentative structure assignment, and relative (%) area of the peaks of the monoclonal antibody produced by CHO-K1 cells under different culture conditions: T-Flask; CYi – Cytodex 3 in vented conical tubes; CYS – Cytodex 3 in shake flask**

			% Area								
Peak	Mean GU^a^	Tentative assignment	T-Flask	CY1	CY2	CY3	CY4	CY5	CY6	CY7	CYS
1	4.9 ± 0.01	A1	2.09	3.33	3.54	3.03	3.76	10.54	4.48	1.77	6.10
2	5.33 ± 0.01	FA1 / A2	2.01	1.75	1.59	2.54	3.03	3.40	1.56	1.74	3.32
3	5.66 ± 0.01	A1G1	-	5.69	5.76	5.15	5.31	14.16	5.70	-	8.60
4	5.80 ± 0.01	**FA2**	**40.99**	**22.01**	**16.59**	**20.59**	**21.24**	**16.93**	**16.87**	**30.92**	**27.57**
5	6.18 ± 0.01	M5 / A2G1	1.50	1.93	1.68	2.44	2.39	3.13	1.77	2.45	2.82
6	6.31 ± 0.04	A2G1[6]	-	1.61	1.19	1.97	2.55	-	1.36	1.29	4.86
7	6.42 ± 0.01	A2G1[3]	-	2.34	2.49	1.82	1.88	6.22	2.77	-	-
8	6.64 ± 0.01	**FA2G1[6]**	**23.52**	**20.74**	**15.60**	**16.12**	**17.10**	**13.20**	**15.62**	**25.59**	**23.46**
9	6.75 ± 0.01	**FA2G1[3]**	**9.11**	**15.18**	**10.53**	**11.45**	**12.98**	**7.33**	**11.77**	**13.23**	**9.85**
10	7.15 ± 0.02	A2G2 / M6	1.65	2.59	2.15	1.94	2.37	3.25	2.72	1.81	2.59
11	7.59 ± 0.01	**FA2G2**	**8.40**	**14.65**	**11.74**	**9.86**	**11.60**	**7.76**	**12.61**	**13.43**	**9.27**
12	7.97 ± 0.01	FA2G1S1	2.73	2.16	2.18	1.91	1.83	2.86	2.54	1.72	1.56
13	8.46 ± 0.01	A2G2S1	0.91	1.29	2.12	2.03	1.58	2.44	1.98	1.09	-
14	8.70 ± 0.02	FA2G2S1[3]	-	-	-	-	-	0.85	1.08	-	-
15	8.88 ± 0.01	FA2G2S1[6]	0.98	1.26	2.38	2.19	1.71	0.78	1.68	1.06	-
16	9.17 ± 0.02	A2G2S2[3]	-	-	-	-	-	-	-	-	-
17	9.36	A3G3S1	-	-	-	-	-	-	-	-	-
18	9.58 ± 0.01	A2G2S2[6]	1.99	0.73	3.70	2.31	1.88	1.95	2.64	0.94	-
19	10.03 ± 0.01	FA2G2S2	0.95	1.25	6.43	5.66	3.17	2.84	4.94	1.54	-
20	10.22 ± 0.00	A3G3S2	-	-	1.18	1.11	0.54	-	1.04	-	-
21	10.43 ± 0.01	FA3G3S2[3]	1.14	1.49	4.56	5.85	3.73	2.35	5.08	1.40	-
22	10.56 ± 0.01	FA3G3S2[6]	-	-	2.05	-	-	-	-	-	-
23	10.68	FA3G3S2[3,6]	0.92	-	-	-	-	-	-	-	-
24	10.93 ± 0.04	A3G3S3	0.84	-	2.54	2.01	1.34	-	1.79	-	-
25	11.12 ± 0.01	A3G3S3[3]	-	-	-	-	-	-	-	-	-
26	11.43	FA3G3S3	0.27	-	-	-	-	-	-	-	-
		Main structures	82.02	72.58	54.46	58.02	62.92	45.22	56.87	83.17	70.15
		Other structures	17.98	27.42	45.54	41.96	37.07	54.77	43.13	16.81	29.85

The main structures are noted in bold.

^a^Average of the GU value of all samples containing the peak.

The reduction of the prevalence of these main structures in microcarrier culture is obviously accompanied by an increase in the remaining structures, particularly of core fucosylated disialylated glycans such as FA2G2S2 (peak 19) and FA3G3S2 (peak 21), and of A1 (peak 1) and A1G1 (peak 3).

 The changes on the mAb glycosylation profile observed between the T-flask and the microcarrier cultures may result from the creation of a different microenvironment for the cells in the microcarriers (Spearman et al. 2005). This may impact glycosylation directly or through variations caused on cell growth characteristics and mAb productivity, such as the ones observed in this study. Effectively, it is clear from Table 2 that microcarriers provide variable cell concentrations, but mAb productivities are always enhanced when compared to T-flasks. Furthermore, it is known that CHO cells produce enzymes such as sialidase, beta-galactosidase, and fucosidase that can accumulate extracellularly in culture and potentially lead to extracellular modifications of the glycans (Gramer and Goochee 1993). By affecting cell characteristics, microcarrier culture may also influence the production of such enzymes and potentially interfere with glycosylation.

**Table 2:** **Data of average cell concentration and productivity of monoclonal antibody-producing CHO-K1 cells cultured in different conditions: T-flask; CYi – Cytodex 3 in vented conical tubes; CYS – Cytodex 3 in shake flask**

Assay	Av. viable cell conc.	***q***_mAb_
	(x10^5^ cells/mL)	(pg/cell/day)
T-flask	8.51 ± 0.06	0.21 ± 0.05
CY1	6.86 ± 1.06	2.57 ± 1.38
CY2	16.02 ± 1.98	0.84 ± 0.35
CY3	3.73 ± 0.63	3.89 ± 1.17
CY4	6.21 ± 0.62	2.16 ± 0.94
CY5	7.63 ± 0.76	1.75 ± 0.66
CY6	18.51 ± 1.08	1.26 ± 0.99
CY7	10.25 ± 0.73	1.51 ± 0.30
CYS	104.1 ± 6.00	1.86 ± 0.58

Furthermore, microcarrier cultures were performed under rocking conditions, which were not present in the typical T-flask cultures, and which may also be a factor leading to the differences found between the glycosylation profile of the mAb obtained in these two types of cultures.

Several studies have focused on the effect of specific elements of the glycan profile on the effector function of glycosylated proteins. Known as functional elements, these include galactose, core fucose, and sialic acid, and have been shown to influence antibody-dependent cellular cytotoxicity (ADCC) and complement-dependent cytotoxicity (CDC). In fact higher levels of galactosylation (Abès and Teillaud 2010; Hodoniczky et al. 2005; Jefferis and Lund 1997; Serrato et al. 2007), reduced core fucosylation (Shields et al. 2002; Shinkawa et al. 2003; Sibéril et al. 2006) or reduced sialylation (Anthony and Ravetch 2010; Anthony et al. 2008; Kaneko et al. 2006; Scallon et al. 2007) are suggested to enhance the clinical efficacy of mAbs that exert their therapeutic effect by ADCC and CDC mediated killing, such as those used for cancer treatment, including the mAb here assessed. This is particularly relevant for core fucosylation, whose absence can improve ADCC activity by up to 100-fold (Shields et al. 2002).

The relative percentages of the main functional elements of the mAb produced under the different culture conditions of this study are shown in Table 3. In a first assessment it is possible to detect a general increase of galactosylation (mainly digalacto (G2) and trigalacto (G3) structures) and decrease of fucosylation in microcarrier cultures compared to T-flasks, both having a potential positive effect on mAb activity. On the other hand, although also diverging from the T-flask cultures, the levels of sialylation in the microcarrier assays are more variable, without showing a common tendency, which indicates that this element may be more susceptible to the culture conditions assayed. As mentioned above, it has been shown that CHO cells produce an extracellular sialidase capable of modifying the sialic acid content of glycoproteins (Gramer and Goochee 1993), and the production/accumulation of this enzyme may be influenced by culture conditions, therefore affecting the degree of sialylation. Additionally, it should be noted that the reported degree of sialylation in human IgG has been below 20% (Arnold et al. 2007; Pučić et al. 2011; Serrato et al. 2007), but some of the microcarrier cultures showed superior levels (e.g. CY2), mainly due to a higher prevalence of disialylated (S2) structures, both digalacto (S2G2) and trigalacto (S2G3), with potential negative effects on mAb activity by reducing ADCC.

**Table 3:** **Galactose, core fucose and sialic acid composition of the monoclonal antibody produced by CHO-K1 cells during microcarrier culture in different conditions: T-flask; CYi - Cytodex 3 in vented conical tubes; CYS - Cytodex 3 in shake flask**

	% Area								
Glycan structures	T-flask	CY1	CY2	CY3	CY4	CY5	CY6	CY7	CYS
Total peaks	17	17	20	19	19	17	20	15	11
Agalactosylated (G0)	46.67	29.35	23.64	28.35	30.41	34.06	25.16	36.56	39.70
Galactosylated (G1+G2+G3)	**53.34**	**70.65**	**76.37**	**71.63**	**69.58**	**65.93**	**74.85**	**63.42**	**60.31**
Monogalactosylated (G1)	36.11	48.69	38.59	39.64	42.85	45.34	40.65	43.06	49.74
Digalactosylated (G2)	14.06	20.48	27.45	23.02	21.13	18.25	26.29	18.97	10.57
Trigalactosylated (G3)	3.17	1.49	10.33	8.97	5.61	2.35	7.91	1.40	0.00
Core fucosylated (F)	89.01	78.74	72.06	73.63	73.36	54.90	72.19	88.89	71.71
Core fucosylated agalactosylated (FG0)	**40.99**	22.01	16.59	20.59	21.24	16.93	16.87	30.92	27.57
Core fucosylated monogalactosylated (FG1)	35.36	**38.08**	**28.31**	**29.48**	**31.91**	**23.39**	**29.93**	**40.54**	**34.87**
Core fucosylated digalactosylated (FG2)	10.33	17.16	20.55	17.71	16.48	12.23	20.31	16.03	9.27
Core fucosylated trigalactosylated (FG3)	2.33	1.49	6.61	5.85	3.73	2.35	5.08	1.40	0.00
Sialylated (S1+S2+S3)	10.73	8.18	27.14	23.07	15.78	14.07	22.77	7.75	1.56
Monosialylated (S1)	4.62	**4.71**	6.68	6.13	5.12	6.93	7.28	3.87	**1.56**
Disialylated (S2)	**5.00**	3.47	**17.92**	**14.93**	**9.32**	**7.14**	**13.70**	**3.88**	0.00
Trisialylated (S3)	1.11	0.00	2.54	2.01	1.34	0.00	1.79	0.00	0.00
Monosialylated monogalactosylated (S1G1)	2.73	2.16	2.18	1.91	1.83	2.86	2.54	1.72	**1.56**
Monosialylated digalactosylated (S1G2)	1.89	**2.55**	4.50	4.22	3.29	4.07	4.74	2.15	0.00
Monosialylated trigalactosylated (S1G3)	0.00	0.00	0.00	0.00	0.00	0.00	0.00	0.00	0.00
Disialylated digalactosylated (S2G2)	**2.94**	1.98	**10.13**	**7.97**	**5.05**	**4.79**	**7.58**	**2.48**	0.00
Disialylated trigalactosylated (S2G3)	2.06	1.49	7.79	6.96	4.27	2.35	6.12	1.40	0.00
Trisialylated trigalactosylated (S3G3)	1.11	0.00	2.54	2.01	1.34	0.00	1.79	0.00	0.00
Complex glycans	98.43	97.74	98.09	97.81	97.62	96.81	97.76	97.87	97.30
High mannose	1.58	2.26	1.92	2.19	2.38	3.19	2.25	2.13	2.71

The major structures are marked in bold.

Hypergalactosylation has been associated with low cell densities in previous studies (Kumpel et al. 1994), but there was no correlation found in this work between the higher levels of mAb galactosylation and the cell densities achieved (Table 2) in microcarrier cultures. For core fucose, it is interesting to note that in addition to the reduction observed in the total levels of fucosylation, variations are also found in the ratio of agalacto:monogalacto:digalacto:trigalacto (FG0:FG1:FG2:FG3) structures, with a general switch from the FG0 prevalence in T-flask cultures to a FG1 prominence in microcarriers. In both cases, the divergences observed between microcarrier and T-flask cultures may have been caused by the use of rocking in the former.

Furthermore, it should be noted that the potentially better levels (in terms of mAb activity) of each of the functional elements are not obtained in the same conditions. For example, in terms of galactosylation, mAb with improved characteristics (higher galactose) was produced in CY2; while for fucosylation, the best quality mAb (less core fucose) was obtained in CY5; and for sialylation, the lower and best levels were obtained in CY1, CY7, and CYS.

It has been suggested that increases in productivity, such as the ones observed in the microcarrier cultures, may potentially lead to decreased effectiveness of recombinant proteins (Nam et al. 2008). However, in this work, the increased productivities of microcarrier cultures (Table 2) do not seem to correlate with a lower quality of the mAb.

In a more specific analysis, the different conditions evaluated for the microcarrier cultures were considered. Rocking speed influences the mAb glycosylation profile, but this effect is highly dependent on the rocking methodology. For example, when using pulse/continuous rocking methodology, 60 rpm enables the production of mAbs with lower levels of sialylation (reduced S2 structures) compared to 40 rpm (CY1 versus CY3), which are potentially more effective through ADCC. However, using a continuous rocking (CY2 versus CY4), these observations are reversed, and 40 rpm (CY4) appears as the advantageous rocking speed. There seems to be no correlation between these results and the cell concentrations and productivities achieved in these assays (Table 2). Nevertheless, the combination of rocking speed and mechanism should be considered for mAb quality.

It is mentioned in the literature that the success of the microcarrier culture is dependent on the initial colonization of the microcarriers with the cells (Blüml 2007; Butler 1996; Voigt and Zintl 1999), so the culture conditions during the first hours of culture should be optimized to improve this initial cell adhesion. In this work, two parameters that have been related to the efficiency of initial cell adhesion were assessed, namely the rocking methodology used for the first six hours of culture and the volume of the culture at inoculation.

Regarding the rocking methodology, it was found that this culture parameter affects the glycosylation profile, but diverges with the concentration of cell inoculum used. Indeed, when using 2 × 10^5^ cells/mL (CY1 versus CY2), continuous rocking resulted in increased levels of sialylation, mainly due to a higher presence of S2 structures, which potentially results in a less efficient mAb due to a decreased ADCC activity. However, when using a cell inoculum of 4 × 10^5^ cells/mL (CY6 versus CY7), the continuous rocking leads to a mAb with lower galactosylation and sialylation, and higher fucosylation, whose biological impact is difficult to predict due to the opposite effects of these functional elements, although a decline of mAb effectiveness would be expected due to the greater impact of fucosylation on mAb functionality. Nevertheless, the combination of rocking methodology at the beginning of the culture and concentration of cell inoculum seems to be important for mAb quality.

Concerning the culture volume, the use of total (5 mL) or half (2.5 mL) volume was tested (CY1 versus CY5), with half (CY5) leading to a higher sialylation (mainly due to increased S2) and to a great reduction in the levels of fucosylation. Due to the known strong impact of fucosylation on IgG activity through ADCC (Shields et al. 2002), it is expected that the positive effects associated with its reduction in these conditions will surpass the negative effects of an increased sialylation, and result in a mAb with improved activity. The use of different volumes at inoculation may affect the microenvironment of the cells and therefore influence glycosylation. Furthermore, although it has been suggested that the use of half volume during the initial hours of culture may improve cell adhesion and consequently the final cell and product yields, such effect was not observed in this study.

In addition to the functional elements already discussed, other less abundant glycoforms may also have an important effect on the biological activity of therapeutic IgGs. The presence of high mannose structures, for example, has been associated with reduced ADCC and CDC (Kanda et al. 2007), as well as with a rapid IgG clearance from serum (Abès and Teillaud 2010). In this study, the levels of high mannose are, in general, slightly increased in microcarrier cultures (particularly for CY5).

The influence of the culture vessel on the glycosylation profile of the mAb produced by CHO cells in microcarriers was also considered in this work, by comparing vented conical tubes with shake flasks. The culture vessel was highly influential particularly in the sialylation levels of the mAb, which abruptly decreased to practically absent sialylation in shake flasks, predicting a positive outcome on mAb biological activity. Additionally, shake flasks seem to provide a less heterogeneity of glycoforms, as demonstrated by the lowest number of peaks obtained (11 peaks, Table 3). Since cell concentrations are highly improved in shake flasks when compared to the vented conical tube cultures, it is possible that these improved levels, or the factors that led to them (e.g. better oxygenation and mass transfer), have caused these modifications on sialylation and heterogeneity.

Some additional cultures were performed with a different type of microcarrier, specifically the macroporous CultiSpher-S, for effects of comparison (data not shown). The global influence of CultiSpher-S cultures on glycosylation, in comparison with T-flask cultures, was similar to that previously observed with Cytodex 3, causing a general reduction on the percentage of main structures, an increase of galactosylation, a slightly reduction of fucosylation, and a variable effect on sialylation. Furthermore, two culture variables tested with CultiSpher-S (initial culture volume and culture vessel) also demonstrated effects on glycosylation similar to those of Cytodex 3 cultures. Of note is the strong impact of the culture vessel on the glycosylation profile obtained with both microcarrier cultures, with shake flasks confirming to be the best option by decreasing the heterogeneity of glycoforms and leading to remarkable decreases of mAb sialylation with CultiSpher-S. The cell densities achieved on these microcarriers were also significantly improved in shake flasks, reinforcing the possibility of this being the cause for the positive changes observed in the glycosylation profile of the mAb.

## Conclusions

An understanding of the role of culture conditions on glycosylation is becoming increasingly important to ensure a consistent quality of the product. In this study, the effect of microcarrier culture and different culture conditions on the glycosylation pattern of a mAb produced by CHO-K1 cells was evaluated. Higher levels of galactosylation (main di- and trigalacto structures) and lower degrees of fucosylation were found in mAb produced in microcarriers when compared to the typical T-flask culture, both potentially improving the mAb effectiveness. On the other hand, sialylation was found to be highly variable without a specific tendency. Furthermore, contrary to what has been suggested, the increased productivities obtained in microcarrier cultures did not correlate with a lower quality of the mAb. On the other hand, the fact that microcarrier cultures are performed under rocking conditions, as opposed to T-flask cultures, and the possible creation of specific microenvironments in the microcarriers may be a cause for the divergences found between them in the glycosylation profile of the mAb produced.

All the culture conditions assessed in microcarrier culture led to modifications on the mAb glycan profile, with each of the functional elements (galactose, core fucose and sialic acid) being divergently affected by these conditions. In particular, the choice of the culture vessel appears to have a strong influence in mAb sialylation, with great and desirable reductions achieved in shake flask cultures. This is potentially related to highly improved cell densities achieved in these vessels, which may have also been the cause for a reduction of the glycoform heterogeneity of the mAb.

## Methods

### Cell line and cell preparation

A CHO-K1 cell line (obtained from American Type Culture Collection) previously transfected for the production of a recombinant human mAb (CAB051 from Biotecnol SA, Lisbon, with application on cancer therapy) was used (Costa et al. 2012). These cells were grown in Dulbecco’s Modified Eagle Medium (DMEM, Sigma-Aldrich) supplemented with 10% fetal bovine serum (FBS, Sigma-Aldrich) and 1 × hypoxanthine-aminopterin-thymidine (HAT, Sigma-Aldrich), at 37°C and 5% CO_2_, in 75 cm^2^ culture flasks (T-flasks). For the microcarrier cultures, cells were detached after reaching 70–80% confluence, and the concentration of the cell suspension adjusted to the values needed for each assay.

### Microcarrier preparation

The microporous Cytodex 3 (Sigma-Aldrich) was used in this work, and prepared according to manufacturer instructions. Briefly, the dry microcarriers were swollen and hydrated in calcium- and magnesium-free phosphate buffered saline (Ca/Mg-free PBS, 137 mM sodium chloride, 2.7 mM potassium chloride, 10 mM sodium phosphate dibasic, and 2 mM potassium phosphate monobasic, pH 7.4, Sigma-Aldrich) for at least 3 h at room temperature, sterilized (121°C, 15 min, 15 psi), and stored at 4°C. Prior to use, the sterilized microcarriers were washed twice with culture medium and once with Ca/Mg-free PBS.

### Microcarrier culture

Microcarrier cultures were initially performed in 50 mL vented conical tubes (INOPAT) and later in 250 mL shake flasks (Sigma-Aldrich).

In vented conical tubes, 5 mL cultures of CHO-K1 cells were performed with 3 g/L of Cytodex 3, and different culture conditions were assessed, as described in Table 4. All cultures were maintained at 37°C and 5% CO_2_, in a support that ensured a fixed 30° inclination angle, placed on an orbital rocking platform. The assays using half the volume of medium at inoculation were completed with the remaining volume after the first 6 h of culture (considered the critical phase for initial cell adhesion). For all assays, the medium was renewed daily by replacing half of the culture volume with fresh growth medium.

**Table 4:** **Conditions tested for the culture of monoclonal antibody-producing CHO-K1 cells in Cytodex 3 microcarriers**

Assay	Inoculum concentration (cells/mL)	Rocking mechanism	Rocking speed (rpm)	Volume at inoculation	Culture vessel
CY1	2×10^5^	Pulse followed by continuous^a^	60	Total	Vented conical tube
CY2	2×10^5^	Continuous	60	Total	Vented conical tube
CY3	2×10^5^	Pulse followed by continuous^a^	40	Total	Vented conical tube
CY4	2×10^5^	Continuous	40	Total	Vented conical tube
CY5	2×10^5^	Pulse followed by continuous^a^	60	Half	Vented conical tube
CY6	4×10^5^	Pulse followed by continuous^a^	60	Total	Vented conical tube
CY7	4×10^5^	Continuous	60	Total	Vented conical tube
CYS	4x10^5^	Pulse followed by continuous^a^	60	Total	Shake flask

^a^ Pulse rocking (60 or 40 rpm for 1 min at each 30 min) during the first six hours of culture, followed by continuous rocking for the remaining time of culture.

In shake flasks, cultures of 20 mL of CHO-K1 cells were performed as described for the vented conical tubes, with the exception of the 30° inclination.

### Culture monitoring

Cell growth was monitored for T-flask and Cytodex 3 cultures. Cells were released from the microcarriers by enzymatic digestion with trypsin (Sigma-Aldrich) and enumerated in a hematocytometer with trypan blue staining (Sigma-Aldrich), for viability assessment. In microcarrier cultures, the cell concentration in the sample was standardized with the number of microcarriers counted microscopically in the sample, and the concentration of cells per microcarrier (C_cell/microcarrier_) determined according to Equation 1.1

Where N_cell_ is the total number of cells counted in the hematocytometer, F the sample dilution factor, V_sample_ the volume of the sample in mL, and N_microcarriers in sample_ the total number of microcarriers counted in the sample.

The cell concentration in the culture (C_cell/mL_) was then determined according to Equation 2.2

Where C_cell/microcarrier_ is the cell concentration per microcarrier, and C_microcarriers/mL_ is the microcarrier concentration in microcarriers per mL as obtained from Equation 3.3

Where C_microcarriers in g/mL_ is the concentration of microcarriers used in the culture in grams of dry weight per mL, and N_microcarriers/g dry weight_ is the approximate number of microcarriers per gram of dry weight (data provided by the manufacturer).

### Antibody quantification

Cell productivity was assessed by enzyme linked immunosorbent assay (ELISA), following an optimized procedure of Biotecnol SA (Portugal). Briefly, dilutions of the samples, a standard of known concentration, and a quality control were added to 96 well plates previously coated with capture antibody. After incubation for 2 h at 37°C, a detection antibody was added and the plates further incubated for 2 h at room temperature. A 3,3^′^,5,5^′^-Tetramethylbenzidine (TMB, Sigma-Aldrich) substrate solution was then added and allowed to react for 10 min, at room temperature, the reaction stopped, and the absorbance read at 450 nm. The concentration of mAb (C_mAb_, in μg/mL) in each sample was determined by Equation 4 and the productivity (q_mAb_, in pg/cell/day) calculated according to Equation 5.4

Where Abs_450_ is the absorbance read at 450 nm, a the estimated response at zero concentration, b the slope factor, c the mid-range concentration, d the estimated response at infinite concentration (parameters determined using the R software, version 2.6.2, from the R Foundation for Statistical Computing).5

Where t is the time of production in days.

### N-glycosylation analysis

The glycosylation profile of the mAb obtained from the T-flask and microcarrier cultures at different conditions was assessed.

#### IgG purification

Prior to IgG purification, samples were concentrated to volumes of 200 μL using 15 mL spin concentrators of 5 kDa molecular weight cutoff (Agilent Technologies). Then, the IgG from the concentrated samples was purified with Protein A spin plates (Thermo Scientific) according to manufacturer instructions, with slight modifications. Briefly, the wells of a spin plate were equilibrated with Binding Buffer (phosphate buffered saline, pH 7.2), sample dilutions (50% v/v in Binding Buffer) were added, and the plates incubated for 30 min with moderate agitation. The resin was then washed with Binding Buffer, and the purified IgG eluted (0.5 M acetic acid, pH 2.5) and neutralized (1 M ammonium bicarbonate, pH 7–8.5). The elutions containing the purified antibody were dried overnight.

#### In gel block immobilization

The dried samples were reduced with a solution containing 13.88 mM sodium dodecyl sulfate (SDS), 12.5 mM Tris (pH 6.6) and 0.05 M dithiothreitol (DTT) for 15 min at 65°C; and alkylated with 100 mM iodoacetamide (IAA) for 30 min in the dark. Then, gel blocks were formed by adding a mixture of 22.5 μL of Protogel 30%, 11.25 μL of 1.5 M Tris (pH 8.8), 1 μL of 35 mM SDS, and 1 μL of 34.7 mM ammonium peroxisulphate solution (APS), and finally adding 1 μL of tetramethylethylenediamine (TEMED) to the samples, letting set for 15 min. The in gel block immobilization of the glycoprotein allows an easier removal of the reducing and alkylating agents, as well as of buffers and environmental contaminants, increasing the efficacy of the enzymatic release of the monosaccharides.

#### N-glycan release and fluorescent labeling

Each gel block was cut into small pieces (≈ 2 mm^2^), washed alternately with acetonitrile and 20 mM sodium bicarbonate (pH 7), and dried in a vacuum centrifuge. For the release of *N*-linked glycans, the dried gel pieces were incubated for 15 min in 50 μL of a PNGase F (Prozyme) solution (4% v/v in 20 mM sodium bicarbonate (pH 7)), and a further 12–16 h at 37°C after adding extra 50 μL of 20 mM sodium bicarbonate. The supernatant was then removed (13,000 rpm, 5 min), and the samples subjected to a series of washes with water and acetonitrile, with a 15 min sonication for each wash. The supernatants obtained were collected and dried in a vacuum centrifuge. The dried samples were redissolved in 20 μL formic acid (1%), incubated for 40 min at room temperature and dried in a vacuum centrifuge.

The released *N*-glycans were labeled for fluorescent detection using the LudgerTag^TM^ 2-aminobenzamide (2AB) Glycan Labeling Kit (Ludger), according to manufacturer instructions. Briefly, 5 μL of the 2AB labeling mix were added to each dried glycan sample, incubated 30 min at 65°C, vortexed, spun down, and incubated for further 90 min.

The excess 2AB label from labeled *N*-glycans was cleaned-up using Normal Phase 1 PhyNexus tips (PhyNexus). Briefly, samples were diluted in 95 μL water and 900 μL acetonitrile. The PhyNexus tips were prepared by a series of ten uptake washes with 95% acetonitrile (v/v in water), 20% acetonitrile (v/v in water) and again 95% acetonitrile. Samples were loaded into the PhyNexus tips through ten in-out cycles, followed by ten uptake washes with 95% acetonitrile. The glycans were then eluted with five uptake rounds of 20% acetonitrile, and the elutions collected and dried in a vacuum centrifuge.

#### Normal phase-high performance liquid chromatography

For normal phase-high performance liquid chromatography (NP-HPLC), each dried sample was ressuspended in 20 μL water and 80 μL acetonitrile. NP-HPLC was performed using a TSKgel Amide-80 3 μm (150 × 4.6 mm) column (Tosoh Bioscience) for 60 min runs, at 30°C, using 50 mM ammonium formate as Solvent A and acetonitrile as Solvent B. The runs were performed on a 2695 Alliance separations module (Waters) with a 2475 multi-wavelenght fluorescence detector (Waters), with excitation and emission wavelengths at 330 and 420 nm, respectively. Conditions of the 60 min method were a linear gradient of 35 to 47% Solvent A over 48 min at a flow rate of 0.48 mL/min, followed by a min at 47 to 100% Solvent A and 4 min at 100% Solvent A, returning to 35% solvent A over 1 min and then finishing with 35% solvent A for 6 min.

The systems were calibrated by running an external standard of 2-AB dextran ladder (2-AB labeled glucose homopolymer) alongside the sample runs.

#### Processing of samples

A fifth-order polynomial distribution curve was fitted to the dextran ladder and used to allocate glucose unit (GU) values from retention times, using Empower GPC software (Waters). Tentative assignment of structures to the peaks was then made by matching the GU values obtained with those in GlycoBase (http://glycobase.nibrt.ie/), and using the known IgG1 profile as a guide. The relative percentage of N-linked glycans was then calculated for agalactosylated (G0), monogalactosylated (G1), digalactosylated (G2), trigalactosylated (G3), galactosylated (G1+G2+G3), core fucosylated (F), core fucosylated agalactosylated (FG0), core fucosylated monogalactosylated (FG1), core fucosylated digalactosylated (FG2), core fucosylated trigalactosylated (FG3), monosialylated (S1), disialylated (S2), trisialylated (S3), sialylated (S1+S2+S3), monosialylated monogalactosylated (S1G1), monosialylated digalactosylated (S1G2), monosialylated trigalactosylated (S1G3) disialylated digalactosylated (S2G2), disialylated trigalactosylated (S2G3), trisialylated trigalactosylated (S3G3), high mannose, and complex glycan structures. The relative percentages were determined by summing the percentage of area of the peaks pertaining to each of the abovementioned structures.
